# Some Suggestions That Aid in Surgical Success

**Published:** 1912-06

**Authors:** R. R. Kime

**Affiliations:** Atlanta, Ga.


### SOME SUGGESTONS THAT AID IN SURGICAL SUCCESS.

By R. R. Kime, M. D., Atlanta, Ga.

Surgical success is not simply completing an operation and preventing death of patient while in hospital.

A capital operation, however skillfully done, is not a surgical success unless really indicated and secures relief sought. Surgical success demands accurate diagnosis, a direct tracing of cause and effect, confirmed by pathology of the living, not of the dead.

The removal of an ovary for neurosis is blind surgery, deserves censure, and results in anything but surgical success. The removal of an appendix through an inch and a half incision and leave behind a diseased gall bladder, gall-stones, or ulcer of duodenum, is to court failure. To stitch up a prolapsed kidney and leave behind a diseased appendix, gall bladder or ulcer of duodenum, is not conducive to surgical success. Neither do I consider the removal of a comparatively healthy appendix or ovary and leaving a freely movable kidney with impaired function that also impairs the functions of the colon, duodenum and stomach, as successful surgery.

Blind surgery of the female internal genital organs makes physical, nervous and mental wrecks. Removal of comparatively healthy ovaries in young and middle-aged women is reckless, unwarranted surgery, and should be condemned by physicians and surgeons. Every physician and surgeon should remember the golden words of Gooddell—"A woman has other organs beside the pelvic organs." No surgical operation of any magnitude should be done except in cases of emergency without a physical examination of patient—a thorough examination of urine and often of other excretions, secretions, blood, etc.

Surgical success depends on accurate diagnosis more than any other one thing.

Chronic surgery means chronic results. A slow, uncertain operator with a bad anaesthetist, weaken the vitality and resisting powers of the patient, favor complications and uncertain results.

Rapid operating consistent with good work, with little manipulation or handling of tissues, especially in abdominal and joint work, aid materially in good results.

Free drainage in fulminating appendicitis and general peritonitis is essential to relieve tension and prevent absorption of septic material. The Fowler position favors drainage, gravitatation of septic material away from diaphragm to the pelvis, where motion and absorption is materially lessened.

To drainage and position add protoclysis properly administered, and we have a trio in the treatment of these cases which no surgeon can afford to omit if he does his duty to his patient.

The judicious use of this trio reverses the results in these cases from 90 to 95% deaths to 90 to 95% recoveries in the hands of the average surgeon and better results with the expert.

Gauze drains in abdominal cavity should not come in contact with the intestine, as it will tend to produce fecal fistula. The gauze in all cases should be wrapped or protected with gutta percha tissue or surrounded with rubber tubing.

The Proctoclysis, beside supplying fluids or salines, can be used as a means of medication to great advantage in many cases —sulphonal, quinine, diuretics and nourishment can be added effectively when indicated and get the effect without disturbing stomach. Uriseptin in the proctoclysis in gall bladder cases acts well, but should not be used in large doses where kidneys are seriousy diseased.

In drainage cases, with abscess cavity, after abdominal cavity is walled off the free use of a mixture of aristol ʒSS—Bis. sub. nit. ʒij, olive oil pure, ℥iv, has given me better results than anything else I have ever used.

It disinfects, lessens sloughing, checks suppuration, makes wound fill up rapidly, with less scar tissue.

In results it is close kin to bismuth paste, and much easier handled.

Before use the bottle must be well shaken each time to secure thorough suspension of the bismuth.

The proper preparatory treatment of many cases before operation lessens complications and adds materially to ultimate success.

The establishment of elimination, relief of toxic conditions and increasing the resisting powers of patient are of material benefit in most patients. In this automobile—aeroplane—electric age the surgeon catches the fad, and rushes the patient through the surgical mill ofttimes without sufficient investigation and discrimination.

It is not simply a surgical operation, but the ultimate recovery of the patient that is desired.

Operations for reflex neurosis and hysteria vanish materially under the sunshine of proper study and investigation of each case.

The pain, disturbed metabolism, dysmenorrhea in young women is starved, overtaxed nature in many cases, calling for a better pabulum and not mutilating surgery.

The ovaries are closely related to the ductless glands of the body and the ductless glands of the body have much to do with the physical development, control of nervous system, increasing resisting powers of body, and developing antitoxic power of the blood.

What such patients need in most cases is relief and prevention of toxic conditions, dieting, physical exercise, lessened nerve tension, correction of abnormal conditions, but not removal of organs so vital to their comfort, welfare, physical development, nervous equilibrium and physiological functions in life. I know it is a much slower process, less brilliant, and often very difficult to do, but that is your duty and my duty as physician and surgeons. Incomplete work is another great handicap to successful surgery.

It is a common thing to hear patients say, "I have friends who have been operated upon and are no better," or "that if I have one operation it means more to follow."

This emphasizes again that each case should be investigated, examined, and studied to know how much and what surgical interference is necessary to give complete relief.

This method will prevent many repeated operations.

Multiple operations at one time in the absence of acute infection, will save much time, expense, suffering and add materially to successful surgery.

Curetting uterus in the presence of tubal disease and adhesions is dangerous unless abdomen is opened and complete work done at the same time.

Removal of an appendix, leaving a large retroverted uterus and prolapsed ovaries without correction, is incomplete work.

A simple retroversion of uterus without complications does not require abdominal section, but where complications exist and abdomen is open, then the round ligaments and anterior layer of broad ligaments should be shortened by method giving least traumatism, also ligaments of ovaries shorten if ovaries are prolapsed.

This improves circulation in pelvic organs and gives greater comfort to patient and may prevent future complications.

So we might continue with other work in the abdomen when once it is opened, but this is sufficient.

In conclusion I desire to emphasize again one point, "never remove an ovary from a woman under thirty-five (35) if it can possibly be avoided."

